# Peptidomimetics Targeting the Amyloidogenicity of Nucleophosmin 1 Mutations in Acute Myeloid Leukemia

**DOI:** 10.1002/cbic.202500306

**Published:** 2025-06-17

**Authors:** Daniele Florio, Sara La Manna, Giada Raffaella Fiore, Valentina Roviello, Giuliano Castellano, Sossio Fabio Graziano, Anna Maria Malfitano, Daniela Marasco

**Affiliations:** ^1^ Department of Pharmacy University of Naples “Federico II” 80131 Naples Italy; ^2^ Department of Agriculture University of Naples “Federico II” 80055 Naples Italy; ^3^ Department of Translational Medical Science University of Naples “Federico II” 80131 Naples Italy

**Keywords:** bioinformatic‐based approaches, enhancers of amyloid aggregation, nucleophosmin 1, point mutations

## Abstract

Nucleophosmin 1(NPM1) is an abundant human protein endowed with many functions where whose dysregulation leads to various cancers and mutations are relevant in acute myeloid leukemia (AML). In the wild‐type form, pentameric NPM1 resides mainly in the nucleolus even if it shuttles toward the cytosol exerting its chaperon function; conversely in AML‐mutated versions, it has mainly a cytoplasmic localization, hence the name NPMc+. All types of AML mutations determine an important amyloid aggregation propensity of the C‐terminal domains (CTD) of NPMc+ and to exploit this amyloidogenicity for therapeutical purposes; herein, this study presents the design and structural and functional investigations of a series of peptides analogs of the sequence of the second helix of the three‐helix bundle of the wt CTD as potential enhancers of amyloid aggregation. Peptides are designed by introducing conservative mutations in the native 264−277 fragment, and their structural features and amyloid propensities are assessed through ThT fluorescence, circular dichroism spectroscopies and scanning electron microscopy. Several “accelerator sequences” are employed in amyloid seeding assays (ASAs): The sequence NPM1_264‐277_ K_267_R, with the single mutation Lys^267^/Arg, exhibits the greater ability to act as a promoter of the amyloid aggregation of NPM1_264‐277_, limiting its toxicity and rescuing cell viability in OCI‐AML3 cells.

## Graphical abstract

Previous studies reveal an unexpected tendency for amyloid aggregation in specific regions of the C‐terminal domain (CTD) of nucleophosmin 1 (NPM1), a nucleolar chaperone involved in acute myeloid leukemia (AML). Herein, several peptide analogs of NPM1_264‐277_ (amyloid core) with conservative substitutions are designed and evaluated through biophysical and cellular assays as potential modulators of amyloid aggregation in AML.

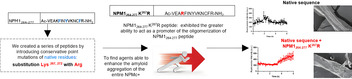

## 1 Introduction

Amyloids can be divided into three main groups:^[^ ^1^ ^]^ i) pathological amyloids,^[^ ^2^, ^3^ ^]^ ii) artificial amyloids both deriving from natural or de novo‐conceived sequences,^[^ ^4^, ^5^, ^6^ ^]^ and iii)functional amyloids which naturally occur and perform a wide range of biological functions in diverse organisms to form bacterial biofilms,^[^ ^7^ ^]^ scaffold for melanin synthesis,^[^ ^8^ ^]^ store peptide hormones,^[^ ^9^ ^]^ form memories,^[^ ^10^ ^]^ and facilitate interactions between proteins in subcellular condensates.^[^ ^11^, ^12^ ^]^ In neurodegenerative mechanisms,^[^ ^13^ ^]^ pathological aggregates can form amorphous assemblies^[^ ^14^ ^]^ and/or highly ordered cross‐β amyloid fibers.^[^ ^15^, ^16^ ^]^ During amyloid aggregation, low‐order or disordered aggregates, as precursors of amyloid fibers, are the most toxic species.^[^ ^17^, ^18^ ^]^ In the identification of novel agents able to suppress the cytotoxicity of amyloids, a recent approach is rising up: Indeed, besides the search for inhibitors of aggregation,^[^ ^19^, ^20^, ^21^, ^22^, ^23^, ^24^, ^25^, ^26^ ^]^ a new interest concerns accelerators of amyloid kinetic and/or enhancers of aggregation which demonstrate capable to convert toxic oligomers into insoluble fibers.^[^ ^27^ ^]^ These substances have often different origins: natural or synthetic^[^ ^28^, ^29^, ^30^ ^]^ or protein fragments.^[^ ^31^, ^32^ ^]^ Interestingly, covalent adducts of diacetyl (DA), a food flavoring, with Aβ_1−42_ peptide transiently increased the neurotoxicity in SH‐SY5Y cells and the Aβ_1−42_ plaque burden as well but decreased neuronal inflammation in transgenic AD mice in vivo.^[^ ^33^ ^]^ Often, mutants of neurodegenerative proteins demonstrated to modulate the native amyloid aggregation: This is the case of human islet amyloid polypeptide (hIAPP) which self‐assembles into amyloid fibrils that deposit in pancreatic islets of type 2 diabetes (T2D) patients. Several small molecules act selectively on its natural variant S20G, which is more amyloidogenic, accelerating its aggregation, interacting, predominantly, with species formed in the lag phase.^[^ ^34^ ^]^

The successful prediction of the aggregation propensity of amino acidic sequences facilitates the investigation of the amyloid processes,^[^ ^35^ ^]^ and the analysis of short amyloid stretches, called aggregation‐prone regions (APRs), represents a reductionistic approach that reveals particularly useful in contrast to experimental complexities deriving from amino acid length and composition, solvent properties, or protein concentration.^[^ ^36^, ^37^ ^]^ The self‐assemblies of APRs are modulated by homo‐ and/or heterotypic interactions as recently demonstrated for Aβ stretches,^[^ ^38^ ^]^ α‐synuclein, and tau K18 proteins where intermolecular associations are largely driven by the electrostatic interaction between the negatively charged C‐terminal segment of α‐synuclein and the positively charged tau K18 fragment.^[^ ^39^ ^]^ Consistently with the amyloid stretch hypothesis, many computational algorithms can be used to predict amyloidogenicity of protein sequence, with different success rates.^[^ ^40^, ^41^, ^42^ ^]^ Cellular functions are often regulated by membraneless organelles composed of proteins and nucleic acids;^[^ ^43^, ^44^ ^]^ these are multicomponent viscoelastic aggregates, with dynamic and reversible assemblages of biomolecules, such as amyloids,^[^ ^45^ ^]^ and are often formed via liquid–liquid phase separation (LLPS). Nucleophosmin (NPM)1 represents the major protein component of the nucleolus^[^ ^46^ ^]^ but is able to transit between the nucleus and cytoplasm and has a chaperone function.^[^ ^47^ ^]^ In NPM1, three distinct regions can be distinguished, each endowed with different functional domains: nucleolar and nuclear localization motifs, nucleic acids binding and oligomerization domains, as well as histones and metal binding regions.^[^ ^48^, ^49^ ^]^ The human N‐terminal domain (NTD) has an eight β‐barrels fold where monomers associate as ring shaped homo‐pentamers.^[^ ^50^ ^]^ NPM1 oligomeric state is tuned by numerous post‐translational modifications, especially phosphorylation regulating protein localization and function.^[^ ^51^ ^]^ The NPM1 central region is intrinsically disordered (IDR) and exhibits highly acidic regions with aspartic and glutamic acids and a nuclear localization signal (NLS).^[^ ^52^, ^53^ ^]^ NPM1 undergoes LLPS either via heterotypic interactions with nucleolar components, as ribosomal RNA (rRNA) and proteins bearing arginine‐rich motifs (Rmotifs) and homotypic interactions among its polyampholytic IDRs.^[^ ^54^, ^55^, ^56^, ^57^ ^]^ The C‐terminal domain (CTD) is characterized by the presence of a basic cluster, followed by a stretch of aromatic region both resulted involved in the recognition of nucleic acids and ATP.^[^ ^58^, ^59^ ^]^ Aromatic residues constitute an atypical nucleolar localization (NoLS), and their mutations are responsible for the unfolding of NPM1 and the aberrant cytoplasmic localization typically observed in acute myeloid leukemia (AML) cases, denoted as NPMc+. Our recent studies highlighted an unexpected propensity of the CTD of NPMc+ to amyloid aggregation, providing cytotoxic species.^[^ ^60^, ^61^, ^62^, ^63^, ^64^, ^65^, ^66^, ^67^, ^68^, ^69^, ^70^ ^]^ AML therapies targeting NPM1 are based on the identification of molecules capable of interfering with its functions as oligomerization, binding to other partners and localization.^[^ ^71^ ^]^ Indeed, AML cells bearing NPM1 mutations retain a certain amount of wt protein into the nucleolus making their nucleolus more vulnerable with respect to cells expressing only wt protein. Thus, novel ways of interfering with the localization and/or oligomerization levels of NPM1 aim to influence its capability to properly build up the nucleolus and, in this context, further enhancing/promoting protein cytoplasmic delocalization could have therapeutic impact.

With the aim to search for agents able to enhance the amyloid aggregation of the entire NPMc+, herein, we analyzed a series of peptides derived from the most amyloidogenic fragment of the protein, NPM1_264‐277_, bearing point conservative mutations of native residues. The tendency of each peptide to aggregate was evaluated through spectroscopic and microscopic techniques. Moreover, the capability of several sequences to act as a seed of aggregation was also assessed and one peptide enhanced the amyloid aggregation of NPM1_264‐277_ fragment.

## 2 Results and Discussion

### 2.1 NPM1_264‐277_‐Mutated Sequences: Design and Bioinformatic Analyses

A series of NPM1_264‐277_ mutations were introduced, by focusing on peculiar substitutions in the amyloidogenic core K^267^−K^273^: The mutation Lys^267^ and Lys^273^ with Arg was introduced leading to the variants NPM1_264‐277_ K^267^R, NPM1_264‐277_ K^273^R, and NPM1_264‐277_ K^267/273^ R reported in **Table** **1** where the T scores from FoldAmyloid server are also reported. The K/R substitution is partially conservative for the common basic nature of side chains even if endowed with distinct steric and hydrogen‐bonding properties. Indeed, the guanidinium group of Arg is bulkier and a stronger hydrogen‐bond donor than the ε‐amino group of Lys. Additionally, we paid attention to the aromatic substitutions Phe^268,276^, Tyr^271^ with, alternatively, Tyr, Phe, and Trp as different aromatics. Hence, the double‐mutated K^267–273^ R was coupled with the single mutations F^268^Y, Y^271^F within the core (NPM1_264−277_ K^267/273^ R, F^268^Y and NPM1_264−277_ K^267/273^ R, Y^271^F) and F^276^Y outside the core (NPM1_264−277_ K^267/273^ R, F^276^Y) and their combinations (NPM1_264−277_ K^267/273^ R, F^268/276^Y, Y^271^F) (Table 1). For the sake of completeness, we introduced also single aromatic mutations F^268^Y (NPM1_264‐277_ F^268^Y), Y^271^F(NPM1_264‐277_ Y^271^F) within the core and F^276^Y (NPM1_264‐277_ F^276^Y) outside the core as well as double mutations F^268^Y−Y^271^F within the core (NPM1_264‐277_ F^268^Y, Y^271^F), Y^271^F‐F^276^Y (NPM1_264‐277 Y271F_,F^276^Y), and F^268^Y‐F^276^Y outside the core (NPM1_264‐277_ F^278/^ ^276^Y); finally, also the triple aromatic mutation was evaluated (NPM1_264‐277_ F^268/276^Y, Y^271^F). These sequences, reported in Table 1, were analyzed using FoldAmyloid to assess their predicted amyloidogenic potential and experimentally evaluated in their aggregative mechanisms. The T score values appear very close to each other, pushing toward an experimental characterization of the propensity to aggregate of individual sequences.

**Table 1:** Sequences, FoldAmyloid score (T score), t_1/2_, and maximum intensity values related to ThT experiments of NPM1_264‐277_ analogs.

Name	Sequence	T score	t_1/2_ (min)	Maximun intensity (a.u.)
NPM1_264‐277_	Ac‐V^264^EAKFINYVKNCFR^277^‐NH_2_	22.00	≈12 ^*^	>1300
NPM1_264‐277_ K^267^R	Ac‐VEARFINYVKNCFR‐NH_2_	22.21	8.01	1284.5
NPM1_264‐277_ K^273^R	Ac‐VEAKFINYVRNCFR‐NH_2_	22.19	11.03	1128.5
NPM1_264‐277_ K^267/273^ R	Ac‐VEARFINYVRNCFR‐NH_2_	22.45	8.02	700
NPM1_264‐277_ K^267/273^ R, F^268^Y	Ac‐VEARYINYVRNCFR‐NH_2_	22.36	Not detected	89.5
NPM1_264‐277_ K^267/273^ R, Y^271^F	Ac‐VEARFINFVRNCFR‐NH_2_	22.54	Not detected	24
NPM1_264‐277_ K^267/273^ R, F^276^Y	Ac‐VEARFINYVRNCYR‐NH_2_	22.36	8.02	452.5
NPM1_264‐277_ K^267/273^ R, F^268/276^Y, Y^271^F	Ac‐VEARYINFVRNCYR‐NH_2_	22.36	Not detected	130
NPM1_264‐277_ F^268^Y	Ac‐VEAKYINYVKNCFR‐NH_2_	21.87	78.01	1163.5
NPM1_264‐277_ Y^271^F	Ac‐VEAKFINFVKNCFR‐NH_2_	22.04	12.01	347
NPM1_264‐277_ F^276^Y	Ac‐VEAKFINYVKNCYR‐NH_2_	21.96	10.0	786
NPM1_264‐277_ F^268^Y, Y^271^F	Ac‐VEAKYINFVKNCFR‐NH_2_	21.95	62.04	347
NPM1_264‐277_ Y^271^F, F^276^Y	Ac‐VEAKFINFVKNCYR‐NH_2_	21.96	2.05	612.5
NPM1_264‐277_ F^268/276^Y	Ac‐VEAKYINYVKNCYR‐NH_2_	21.78	18.10	147
NPM1_264‐277_ F^268/276^Y, Y^271^F	Ac‐VEAKYINFVKNCYR‐NH_2_	21.87	6.03	239.5

### 2.2 Kinetic of Aggregation: Distinct Aggregation Propensities of NPM1_264‐277_ Analogs

**Figure 1:**
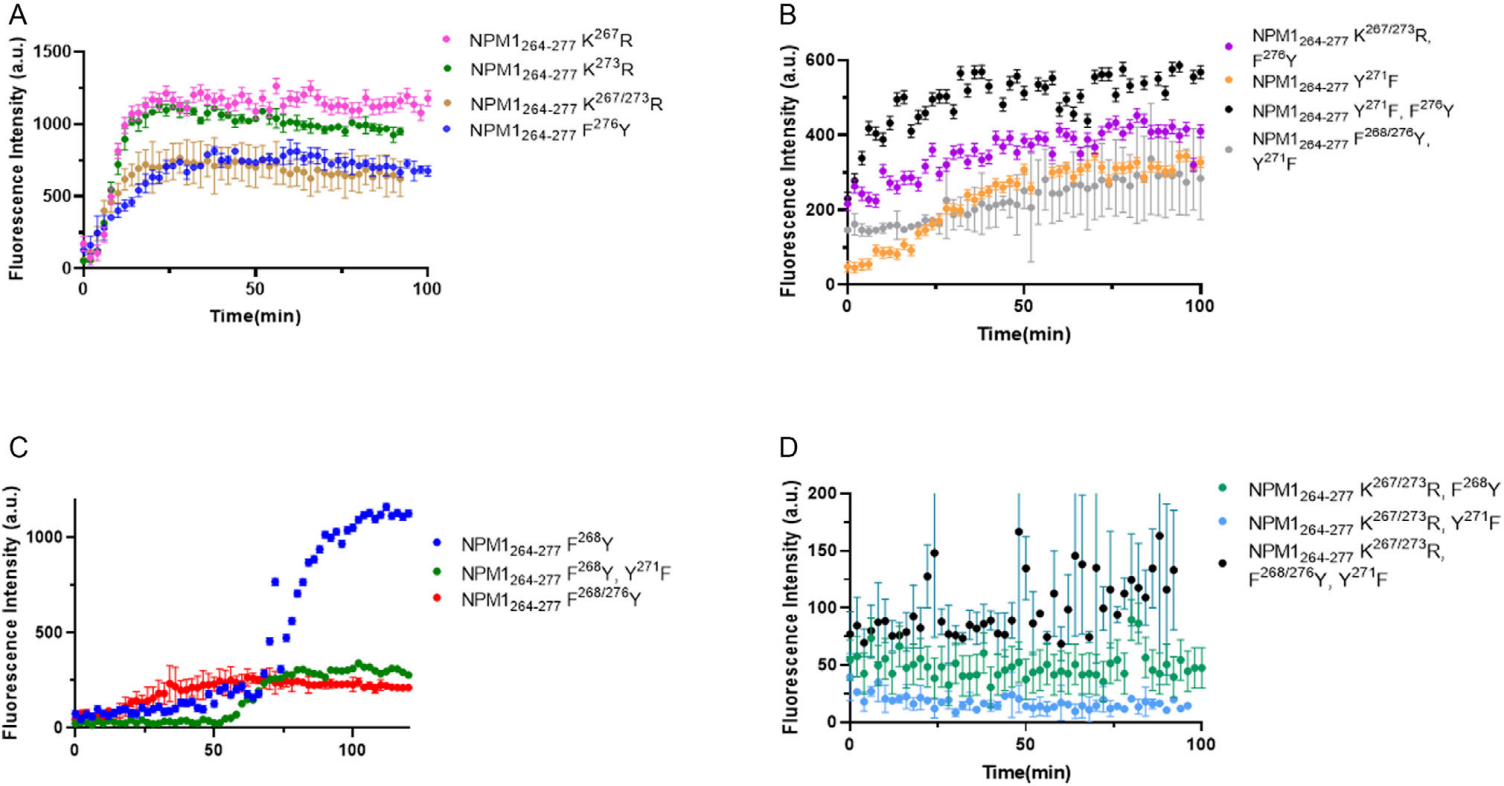
Time courses of ThT fluorescence emission intensity of indicated peptides belonging to A) A, B) B, C) C, and D) D groups. Error bars are standard deviations from two independent experiments.

To evaluate the tendency to aggregate of NPM1_264‐277_ analogs, Thioflavin T (ThT) assay was carried out, and the overlay of time courses profiles is reported in **Figure** **1**. Since peptide sequences exhibited very different behaviors and to properly compare them also, the time values at which ThT fluorescence emissions reach their maximum value/2, named t_½_, and fluorescence maxima values were evaluated (Table 1). Despite an extensive HFIP treatment, NPM1_264‐277_ peptide is quickly prone to aggregation even at t = 0 of analysis, without a sufficient lag‐phase as reported for similar systems.^[^ ^72^ ^]^ However, its kinetic ThT profile is reproducible,^[^ ^73^, ^74^, ^75^ ^]^ even if it allows only a rough estimation of t_1/2_ value of 12 min (Figure S2, Supporting Information). To rationalize kinetic profiles, the NPM1_264‐277_ analogs peptides were divided into four groups A−D: The first two groups contain NPM1_264‐277_ analogs having t_1/2_ values ≤ 12 min, which are further subdivided in sequences with intensity values major of 500 a.u., group A (Figure 1A), and those with lower maxima values, group B (Figure 1B). Group C exhibited delayed t_½_ values (>12 min, Figure 1C), while the last group, D, exhibited not significant signal variations. Single mutated sequences, i.e., NPM1_264‐277_ K^267^R and NPM1_264‐277_ K^273^R, NPM1_264‐277_ F^276^Y and the di‐mutated sequence NPM1_264‐277_ K^267/273^ R belong to group A while to group B belong to the tri‐mutated sequences NPM1_264‐277_ K^267/273^ R, F^276^Y and NPM1_264‐277_ F^268/276^Y, Y^271^F and the di‐mutated NPM1_264‐277_ Y^271^F, F^276^Y and the single mutated sequence NPM1_264‐277_ Y^271^F. Into the delayed group C, three sequences bearing aromatic substitution as NPM1_264‐277_ F^268^Y, NPM1_264‐277_ F^268^Y, Y^271^F and NPM1_264‐277_ F^268/276^Y were included. As expected, peptides unable to aggregate (group D) (Figure 1D) are those bearing multimutations as NPM1_264‐277_ K^267/273^ R, F^268^Y, NPM1_264‐277_ K^267/273^ R, Y^271^F, and NPM1_264‐277_ K^267/273^ R, F^268/276^Y, Y^271^F.

### 2.3 Time‐Dependent CD Analysis Reveals Distinct Conformational Features in NPM1_264‐277_ Analogs

**Figure 2:**
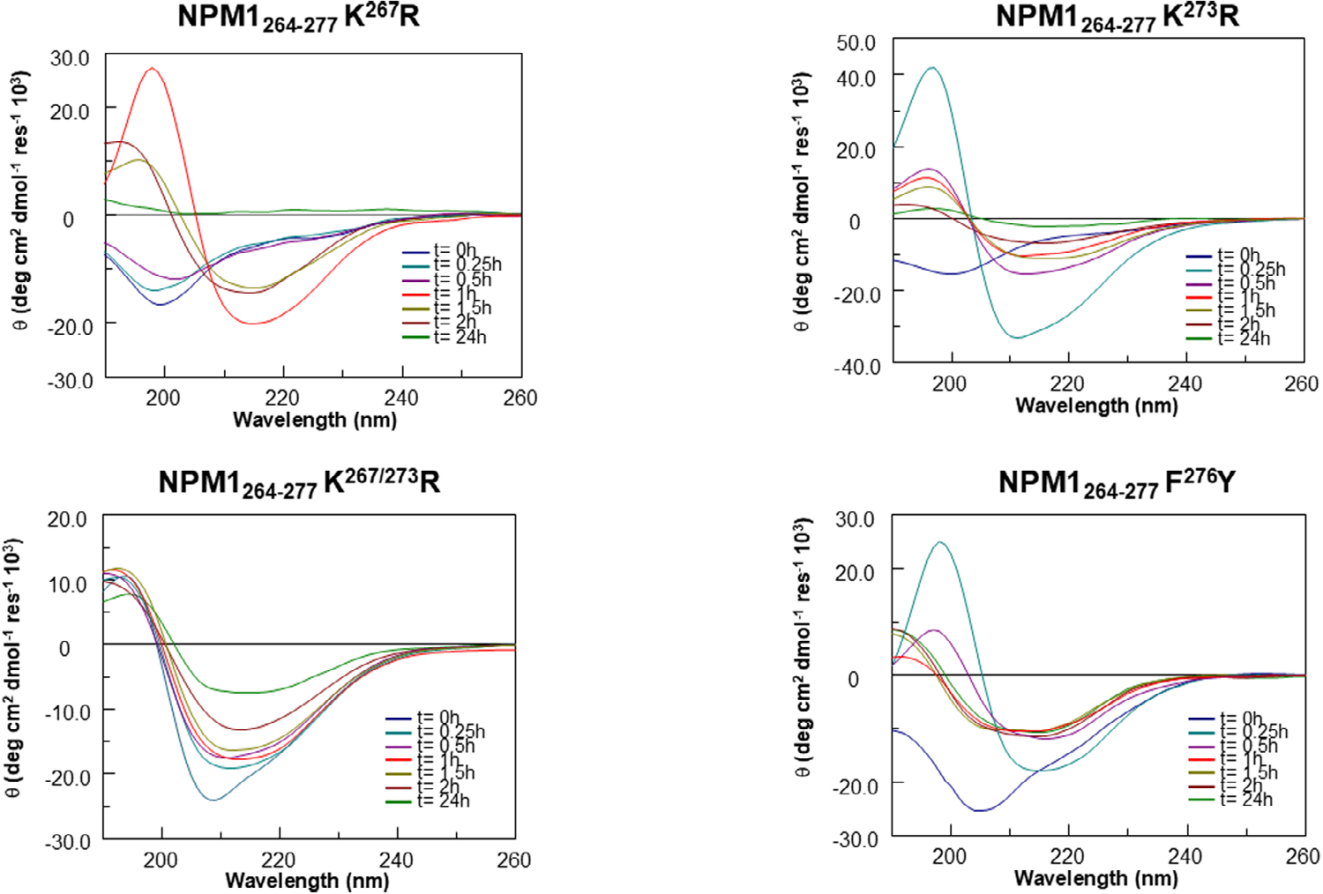
Overlay of CD spectra over time of R‐peptides: NPM1_264‐277_ K^267^R−NPM1_264‐277_ K^273^R−NPM1_264‐277_ K^267/273^ R−NPM1_264‐277_ F^276^Y.

**Figure 3:**
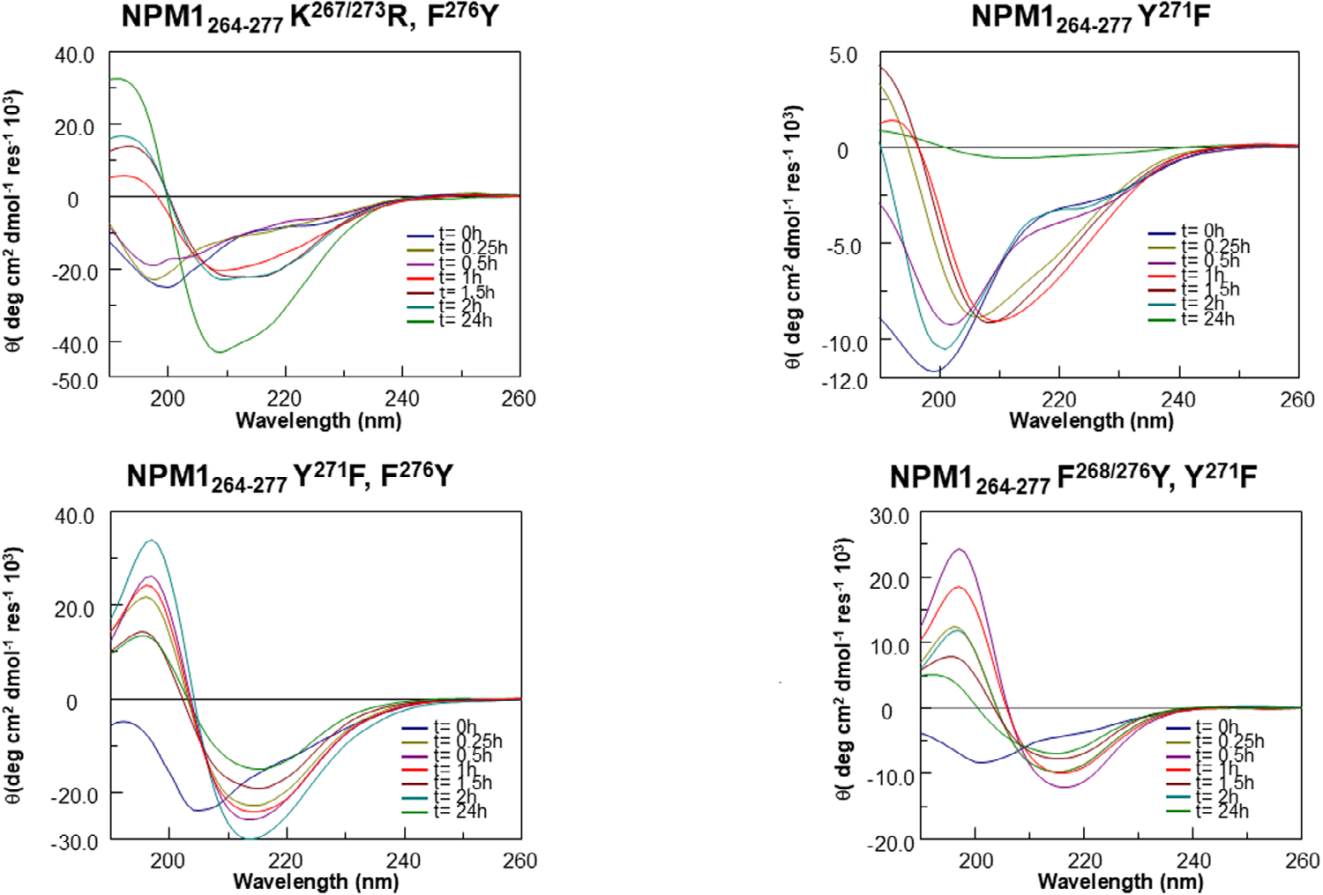
Overlay of CD spectra over time of R‐peptides: NPM1_264‐277_ K^267/273^ R, F^276^Y−NPM1_264‐277_ Y^271^F−NPM1_264‐277_ Y^271^F, F^276^Y−NPM1_264‐277_ F^268/276^Y, Y^271^F.

**Figure 4:**
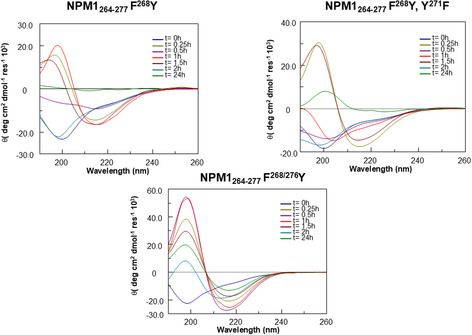
Overlay of CD spectra over time of R‐peptides: NPM1_264‐277_ F^268^Y−NPM1_264‐277_ F^268^Y, Y^271^F−NPM1_264‐277_ F^268/276^Y.

**Figure 5:**
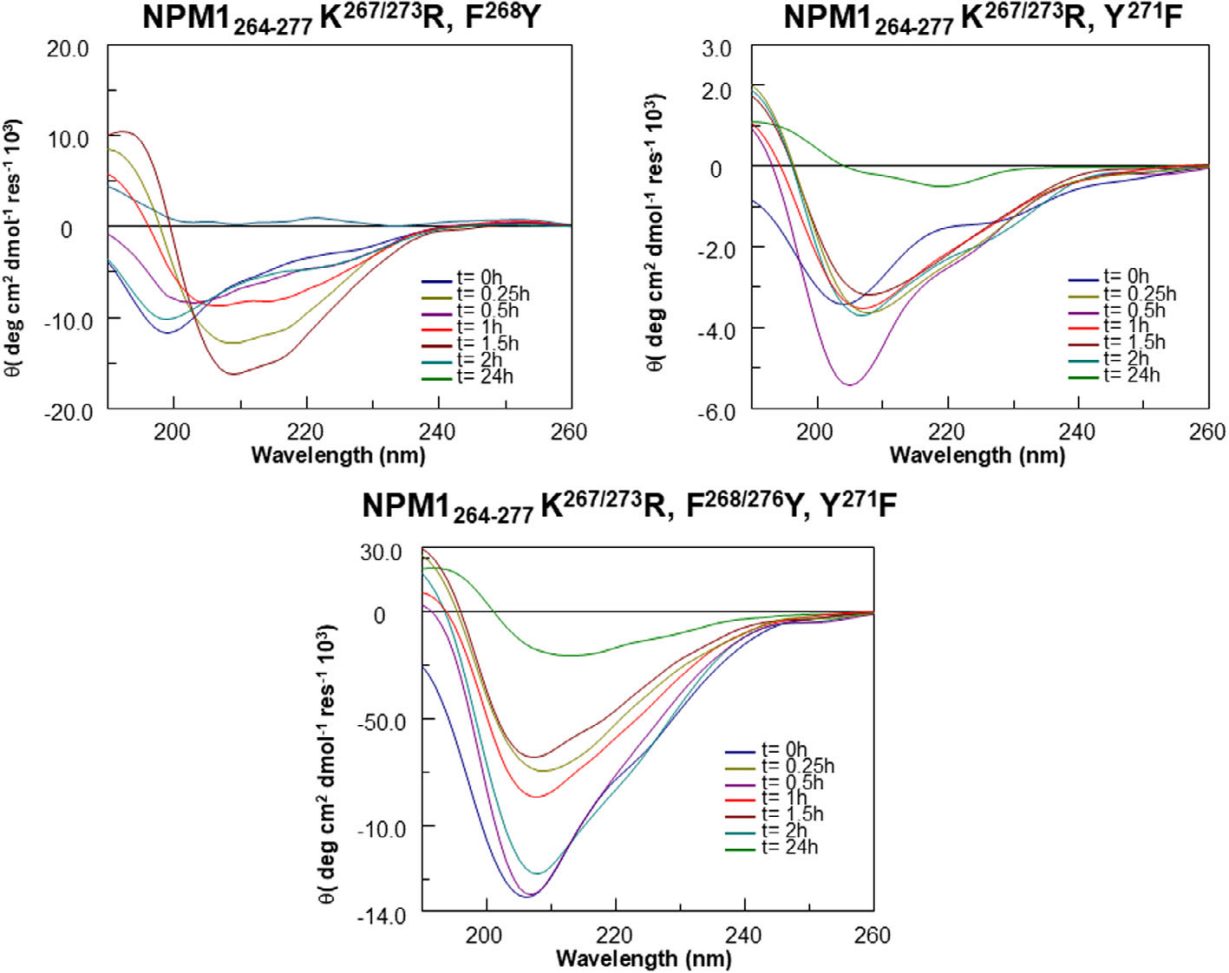
Overlay of CD spectra over time of R‐peptides: NPM1_264‐277_ K^267/273^ R, F^268^Y−NPM1_264‐277_ K^267/273^ R, Y^271^F−NPM1_264‐277_ K^267/273^ R, F^268/276^Y, Y^271^F.

The conformational behavior of NPM1_264‐277_ analogs was followed by circular dichroism (CD) spectroscopy, over time (**Figure** **2**−**5**). We analyzed the behaviors of groups A−D separately, and in Table S1, Supporting Information, the deconvolution of CD data is reported. At the outset of aggregation (t = 0), the 14 NPM1_264‐277_ analogs exhibited a wide range of conformations, and, as the aggregation progressed, peculiar structural changes were observed among the groups. To better evaluate different behaviors, we also reported CD variations of minimum and wavelengths variations versus time, for each sequence. For instance, in group A, NPM1_264‐277_ K^267^R and NPM1_264‐277_ K^273^R initially exhibited a random profile which transited toward β‐sheet in different times (Figure 2). In detail, NPM1_264‐277_ K^267^R exhibited the maximum value of CD signal, close to maximum β content (Table S1, Supporting Information) after 1 h (Figure S3A, Supporting Information (NPM1_264‐277_ K^267^R a)), while NPM1_264‐277_ K^273^R underwent to transition in less time, t = 0.5 h, before the progressive reduction of Cotton effect (Figure S3A, Supporting Information (NPM1_264‐277_ K^273^R a)). NPM1_264‐277_ F^276^Y exhibited more ordered initial conformations (λ_min_ > 200 nm) and after, a transition toward β (Table S1, Supporting Information) and a simultaneous reduction of signal, after t = 0.5 h, which remained constant in the rest of time interval (Figure S3A, Supporting Information (NPM1_264‐277_ F^276^Y a)). NPM1_264‐277_ K^267/273^ R peptide (Figure 2) exhibited a profile quite like that of NPM1_264‐277_ wt with an initial mixed α + β conformation tendent toward β‐structures which appeared stable at longer times (t = 24 h dashed line).

Also, sequences belonging to the B group (Figure 3) exhibited a transition toward β‐conformation, albeit with varying kinetics. While NPM1_264‐277_ Y^271^F reached β‐structure by 1 h, (Figure S3B, Supporting Information (NPM1_264‐277_ Y^271^F a)), NPM1_264‐277_ Y^271^F, F^276^Y and NPM1_264‐277_ F^268/276^Y, Y^271^F rapidly (t = 0,5 h (Figure S3B, Supporting Information (NPM1_264‐277_ Y^271^F, F^276^Y, NPM1_264‐277_ F^268/276^Y, Y^271^F a)) reached high content of β‐structure (Table S1, Supporting Information). Notably, NPM1_264‐277_ K^267/273^ R, F^276^Y exhibited strongly delayed transition toward ordered mixed conformations, α + β, even if it was maintained at longer times (Figure S3B, Supporting Information (NPM1_264‐277_ K^267/273^ R, F^276^Y a)). This behavior was partially shown also by NPM1_264‐277_ Y^271^F, F^276^Y and NPM1_264‐277_ F^268/276^Y, Y^271^F, while NPM1_264‐277_ Y^271^F appeared completely aggregated at t = 24 h.

Peptides belonging to Group C (Figure 4) showed mixed behaviors: NPM1_264‐277_ F^268/276^Y was quite like NPM1_264‐277_ Y^271^F, F^276^Y and NPM1_264‐277_ F^268/276^Y, Y^271^F (Figure S4A, Supporting Information (NPM1_264‐277_ F^268/276^Y a‐b)), while NPM1_264‐277_ F^268^Y and NPM1_264‐277_ F^268^Y, Y^271^F demonstrated unique profiles. NPM1_264‐277_ F^268^Y showed a quick (t = 0.5 h) transition toward ordered conformations partially reinforced during time (Figure S4A, Supporting Information (NPM1_264‐277_ F^268^Y a‐b)), while NPM1_264‐277_ F^268^Y, Y^271^F a partial reduction of Cotton effect and then the transition (t = 1.5 h) toward secondary structures (Figure S4A, Supporting Information (NPM1_264‐277_ F^268^Y, Y^271^F a‐b)).

In group D (Figure 5), NPM1_264‐277_ K^267/273^ R, F^268^Y initially exhibited a random coil profile and then (after 1 h) a β + α‐helix transition with higher α‐helical content (Figure S4B, Supporting Information (NPM1_264‐277_ K^267/273^ R, F^268^Y a‐b)). On the other hand, NPM1_264‐277_ K^267/273^ R, Y^271^F and NPM1_264‐277_ K^267/273^ R, F^268/276^Y, Y^271^F showed similar progression of profile during time as indicated by a minimum at around 205 nm and a gradual decrease in the Cotton effect, indicating aggregation (Figure S4B, Supporting Information (NPM1_264‐277_ K^267/273^ R, Y^271^F, NPM1_264‐277_ K^267/273^ R, F^268/276^Y, Y^271^F a‐b).

### 2.4 SEM Analysis Reveals Distinct Morphological Features of Selected Mutants of NPM1_264‐277_ Aggregates

**Figure 6:**
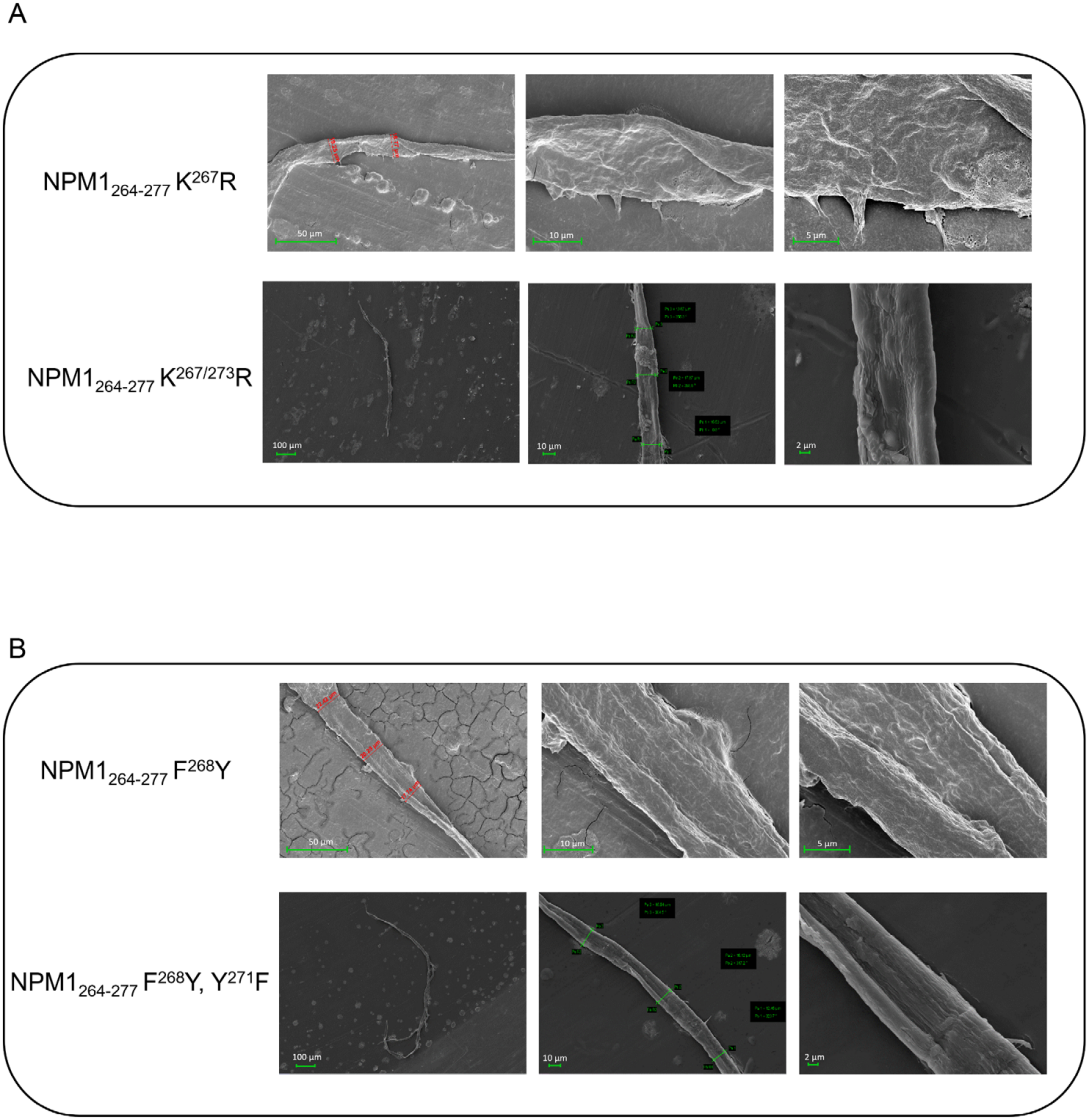
SEM micrographs after 4 h of aggregation of A) NPM1_264‐277_ K^267^R and NPM1_264‐277_ K^267/273^ R and B) NPM1_264‐277_ F^268^Y and NPM1_264‐277_ F^268^Y, Y^271^F. Overviews of the surface of samples at 50 μm (left column), 10 μm (central column), and 5 μm (right column) for R1 and NPM1_264‐277_ F^268^Y and 100 μm (left column), 10 μm (central column), and 2 μm (right column) for NPM1_264‐277_ K^267/273^ R, NPM1_264‐277_ F^268^Y, Y^271^F.

Scanning electron microscopy (SEM) was employed to evaluate potential differences in the morphologies of peptide aggregates. To this purpose, in the group A, NPM1_264‐277_ K^267^R and NPM1_264‐277_ K^267/273^ R were analyzed: They exhibited enhancing amyloid effects in ThT assay. For the very low intensities exhibited in the same analysis, peptides belonging to group B were excluded from SEM studies, while for group C, NPM1_264‐277_ F^268^Y and NPM1_264‐277_ F^268/276^Y were chosen and for group D NPM1_264‐277_ K^267/273^ R, F^268^Y. SEM images, recorded after 4 h of aggregation (**Figure** **6**), revealed distinctive morphological features of aggregated peptides. They exhibited differences in length and width (**Table** **2**) also with respect to NPM1_264‐277_ wt (Figure S5A, Supporting Information): NPM1_264‐277_ K^267^R peptide (Figure 6A) showed a longer and thicker fiber, with respect to NPM1_264‐277_ K^267/273^ R, which appears to be shorter and slightly thinner with a diameter of 16 μm. Among group C members (Figure 6B), the fiber of NPM1_264‐277_ F^268^Y, Y^271^F presented turns to make it longer than NPM1_264‐277_ F^268^Y. NPM1_264‐277_ K^267/273^ R, F^268^Y and NPM1_264‐277_ F^268/276^Y peptides showed no fibers formation during the observed time interval (Figure S5C,D, Supporting Information).

**Table 2:** Diameters and lengths of fibers for the indicated sequences obtained by SEM analysis.

	**Diameter (μm)**	**Length (μm)**
**NPM1**_**264**‐**277**_ a)	15	2099
**NPM1**_**264**‐**277 **_ **K** ^**267**^ **R**	18	613
**NPM1**_**264**−**277**_ **K** ^**267/273**^ **R**	16	196
**NPM1**_**264**−**277**_ **K** ^**267/273**^ **R, F** ^**268**^ **Y**	*b)	*b)
**NPM1**_**264**‐**277**_ **F** ^**268**^ **Y**	20	628
**NPM1**_**264**‐**277**_ **F** ^**268**^ **Y, Y** ^**271**^ **F**	14	1377
**NPM1**_**264**‐**277**_ **F** ^**268/276**^ **Y**	*b)	*b)

a) In agreement with already reported;^[^ ^32^ ^]^

b) * = No fiber.

### 2.5 Amyloid Seeding Assays: NPM1_264‐277_ K^267^R and NPM1_264‐277_ F^268^Y Peptides Demonstrate Distinct Cooperative Behaviors in Modulating the Aggregation Kinetics of Both the wt NPM1_264‐277_ and Cterm_mutA variant

**Figure 7:**
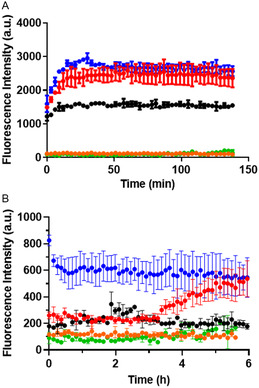
ASA experiments of A) NPM1_264‐277_ alone (black) and in presence of NPM1_264‐277_ K^267^R (blue) and NPM1_264‐277_ F^268^Y (red) seeds and B) Cterm_mutA alone (black) and in presence of NPM1_264‐277_ K^267^R (blue) and NPM1_264‐277_ F^268^Y (red) seeds. As control, NPM1_264‐277_ K^267^R (orange) and NPM1_264‐277_ F^268^Y (green) are reported. Error bars are standard deviations from two independent experiments.

To gain insights into potential cooperation given by designed peptides to the amyloid aggregation of the AML‐CTD, amyloid seeding assays (ASAs) were carried out. We performed these assays on two amyloid sequences coming from NPM1: i) the fragment 264‐277 and ii) the entire CTD in the type A mutated form, Cterm_mutA, by adding two selected sequences, NPM1_264‐277_ K^267^R and NPM1_264‐277_ F^268^Y at the ratio of 1: 0.2. This ratio was chosen as the most suitable to observe only seeding and not co‐assembling effects. These seeding agents were added as pre‐aggregates at the beginning of ThT analysis, and time course profiles are reported in **Figure** **7**. The addition of NPM1_264‐277_ K^267^R to NPM1_264‐277_, enhanced the aggregative process (**Table** **3**, Figure 7A). Similarly, but in a slower way, NPM1_264‐277_ F^268^Y seed accelerated NPM1_264‐277_ aggregation (Figure 7A).

**Table 3:** The *t*_1/2_ and maximum intensity values derived from ASA experiments through ThT assays.

Name	t_1/2_	Maximum intensity (a.u.)
NPM1_264‐277_ + NPM1_264‐277_ K^267^R as seed	6.07 min	2464
NPM1_264‐277_ + NPM1_264‐277_ F^268^Y as seed	9.06 min	2950
Cterm_mutA	Not detected	250
Cterm_mutA + NPM1_264‐277_ K^267^R as seed	4.31 h	536
Cterm_mutA + NPM1_264‐277_ F^268^Y as seed	Not detected	613

**Figure 8:**
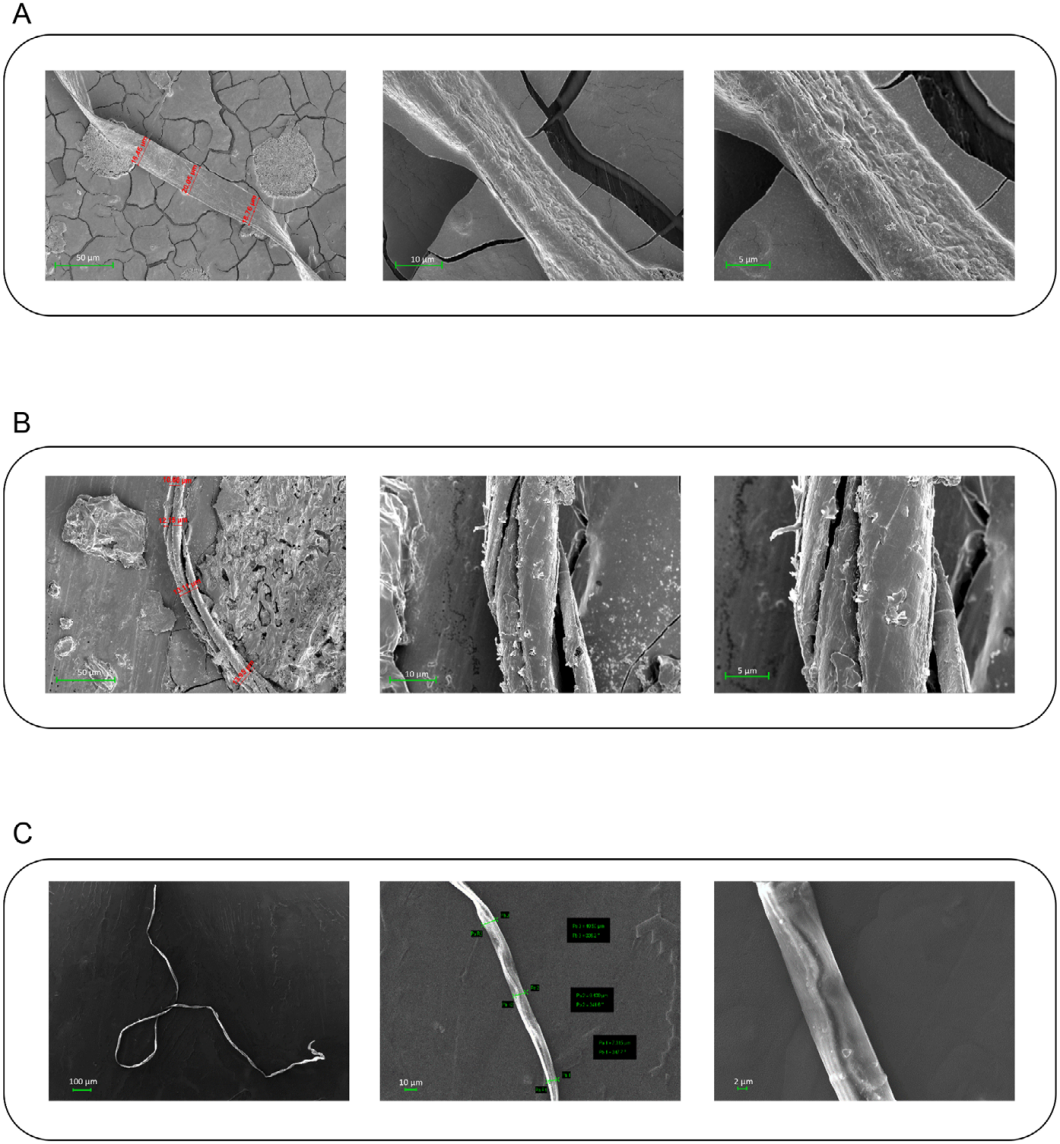
SEM micrographs of ASA experiments of A) NPM1_264‐277_ + NPM1_264‐277_ K^267^R as seed after 4 h of aggregation, B) Cterm_mutA + NPM1_264‐277_ K^267^R, and C) Cterm_mutA + NPM1_264‐277_ F^268^Y as seed after 48 h of aggregation. Overviews of the surface of samples at 50 μm, 10 μm, and 5 μm for (A) and (B); 100 μm, 20 μm and 2 μm for (C).

Conversely, NPM1_264‐277_ K^267^R and NPM1_264‐277_ F^268^Y as seeds demonstrated different effects on Cterm_mutA aggregation: NPM1_264‐277_ K^267^R induced faster aggregation than the polypeptide alone, while NPM1_264‐277_ F^268^Y induced an increase of aggregation only after 4 h (Figure 7B). As controls, NPM1_264‐277_ K^267^R and NPM1_264‐277_ F^268^Y alone pre‐aggregated at “seed concentration” did not exhibit any aggregation. The effect of NPM1_264‐277_ K^267^R on the morphology of fiber deriving from NPM1_264‐277_ did not cause great changes (**Figure** **8**A, **Table** **4**). Conversely, both NPM1_264‐277_ K^267^R and NPM1_264‐277_ F^268^Y caused an elongation of the fibers of Cterm_mutA peptide (Figure 8B,C).

**Table 4:** Diameters and lengths of fibers derived from ASA experiments through SEM analysis.

	**Diameter (μm)**	**Length (μm)**
**NPM1**_**264**‐**277**_ + **NPM1**_**264**‐**277**_ **K** ^**267**^ **R as seed**	19	1084
**Cterm_mutA**	15	869
**Cterm_mutA + NPM1**_**264**‐**277**_ **K** ^**267**^ **R as seed**	12	1031
**Cterm_mutA + NPM1**_**264**‐**277**_ **F** ^**268**^ **Y as seed**	9	1764

### 2.6 DLS and FT‐IR Analyses Underscore the Seeding Capacity of NPM1_264‐277_ K^267^R Peptide in Modulating the Aggregation Behavior of the Cterm_mutA Variant

**Figure 9:**
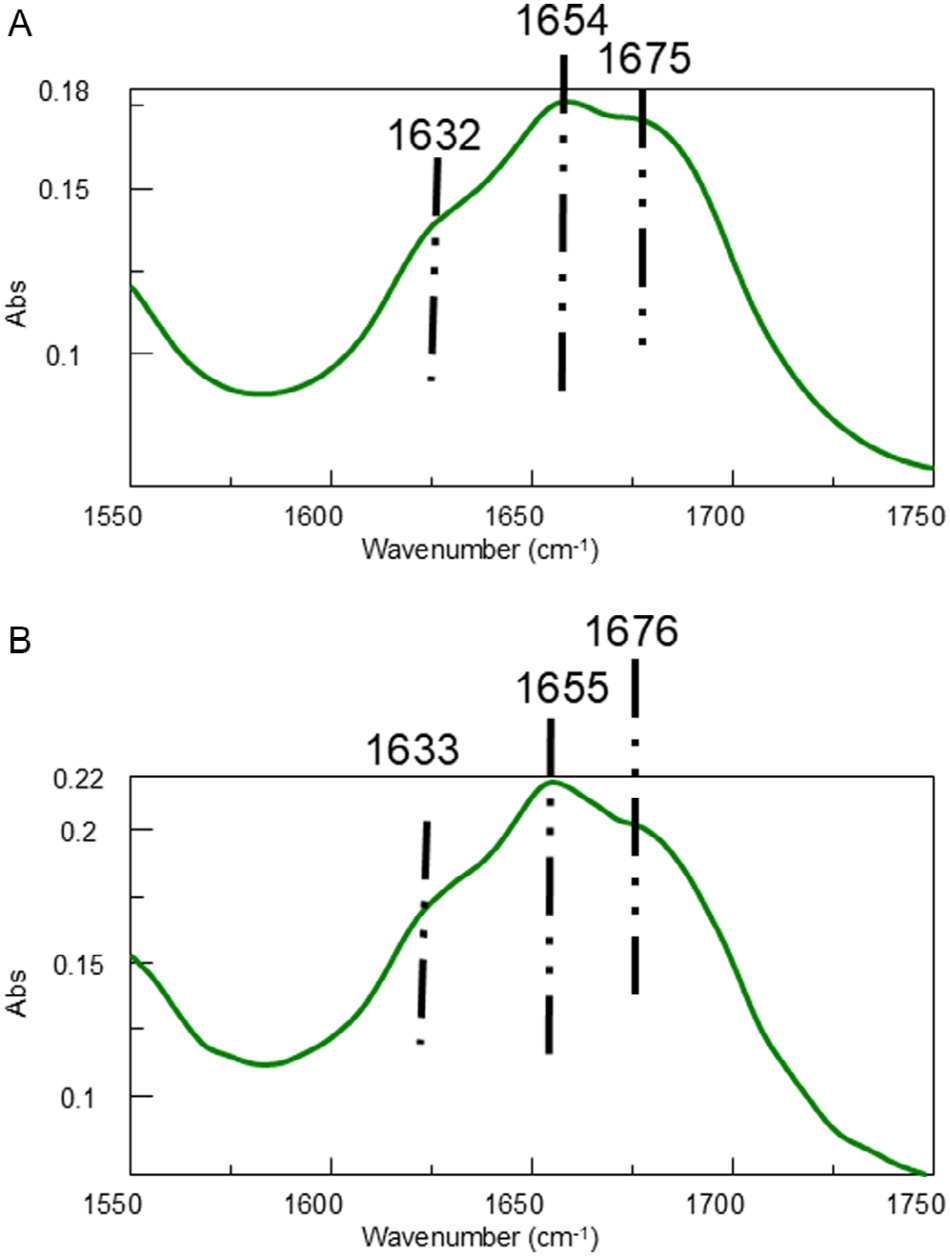
FT‐IR spectra of A) Cterm_MutA and B) Cterm_MutA + NPM1_264‐277_ K^267^R as seed at the molar ratio of 1: 0.2.

Dynamic light scattering (DLS) analysis over time was employed to evaluate possible effects of NPM1_264‐277_ K^267^R as seed on Cterm_mutA. Differently from Cterm_mutA alone, which demonstrated a correct autocorrelation after 17 h of stirring,^[^ ^66^ ^]^ the presence of NPM1_264‐277_ K^267^R as a seed resulted in the formation of a turbid suspension already after 2 h of aggregation (Figure S6A, Supporting Information) which suggested the formation of large aggregates more insoluble with respect to those formed by Cterm_mutA alone, thus confirming a seeding action exerted by the NPM1_264‐277_ K^267^R peptide. Once centrifuged the suspension, the supernatant was observed during time (till 120 h) but never autocorrelation was observed. Conversely, NPM1_264‐277_ demonstrated unable to autocorrelate within 5 h (data not shown). To gain insights into this mechanism, FT‐IR spectroscopy was employed. The absorbance deconvolution in the amide region I of Cterm_mutA (**Figure** **9**A) suggests the presence of β‐sheet arrangements (peak at 1632 cm^−1^) and that these arrangements have an antiparallel orientation within the assemblies, as indicated by the weak band centered at 1675 cm^−1^. We also evaluate the ASA sample obtained using NPM1_264‐277_ K^267^R as seed (Figure 9B): The presence of NPM1_264‐277_ K^267^R did not affect the positions of bands confirming an antiparallel arrangement and suggesting that the observed enhancement effect is likely due to a proper intercalation of NPM1_264‐277_ K^267^R within the β‐sheets aggregates.

### 2.7 MTT Assay: A Partial Rescue of Cellular Viability Upon Treatment with NPM1_264‐277_ K^267^R Peptide as a Preformed Seed under Genotoxic Stress Conditions Suggests a Protective Shift in the Aggregation Landscape

**Figure 10:**
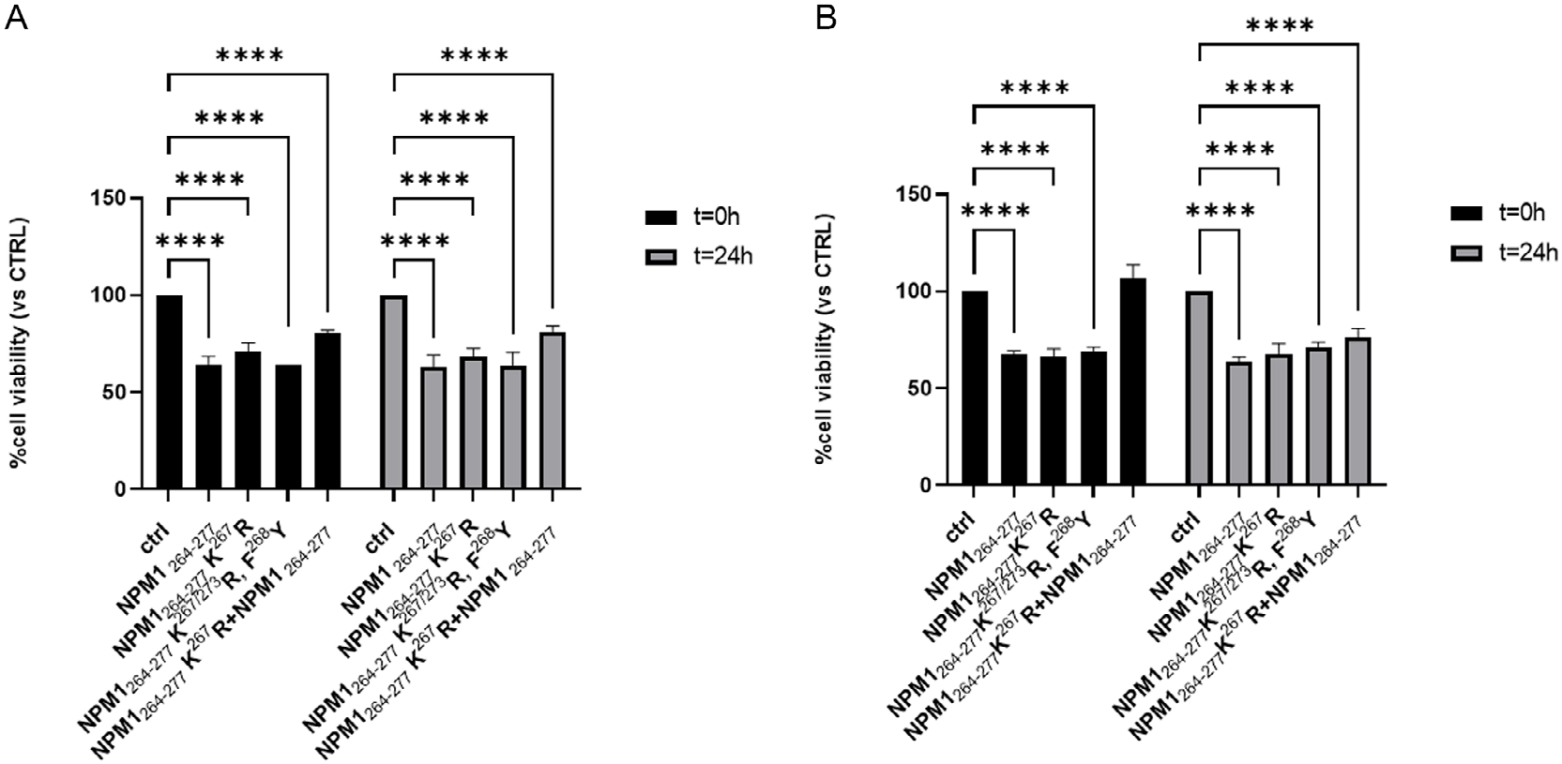
Cell viability effects of NPM1_264‐277_ K^267^R, NPM1_264‐277_ K^267/273^ R, F^268^Y, NPM1_264‐277_ and NPM1_264‐277_ K^267^R in combination with NPM1_264‐277_. The histograms report the values of %cell viability (vs. CTRL) using OCI‐AML3 cells A) untreated or B) pretreated with H_2_O_2_ in the presence or in the absence (ctrl) of the indicated peptides pre‐incubated at two different times: t 0 h and t24h. The histograms are representative of a single experiment performed in triplicate. Results are reported as mean ± SD. The statistical analysis was performed by GraphPad Prism 9 software using two‐way ANOVA corrected for multiple comparison by the Dunne*t* test (****p < 0.0001).

To evaluate different cellular effects due to the creation of large size aggregates upon seeding effect, we employed NPM1_264‐277_ K^267^R as enhancer of aggregation and NPM1_264‐277_ K^267/273^ R, F^268^Y as negative control in cell viability assays in OCI‐AML3 cells.^[^ ^66^ ^]^ Histograms of MTT assay are reported in **Figure** **10**. Different experimental conditions were evaluated: Cells were pretreated (Figure 10A) or not (Figure 10B) with H_2_O_2_ to evaluate genotoxic stress conditions^[^ ^32^ ^]^ and peptides were added to cells at two different times of aggregation (t = 0 and 24 h). NPM1_264‐277_ K^267^R and NPM1_264‐277_ K^267/273^ R, F^268^Y sequences alone exhibited a behavior like that exhibited by NPM1_264‐277_ with a decrease of cell viability of ≈32%, already at t = 0, as expected. Conversely, ASA samples, NPM1_264‐277_ K^267^R + NPM1_264‐277_ (containing NPM1_264‐277_ K^267^R, as seed), exhibited a rescue of cell viability already at t = 0 h in a more evident way in H_2_O_2_ pretreated cells. This is likely due to an enlargement of aggregates, due to seeding effects, which resulted in less toxicity. This effect however disappeared at 24 h. As expected, the other seed sample NPM1_264‐277_ K^267/273^ R, F^268^Y + NPM1_264‐277_ (containing NPM1_264‐277_ K^267/273^ R, F^268^Y as seed) did not produce any enhancement of cell viability.

## 3 Conclusion

In this work, by following a protein‐based approach, we pursued a hypothesis on NPMc+ accumulation: The amyloid aggregation could likely concur to the formation of cellular aggregates, and since these aggregates are cytotoxic, a further induction of aggregation by external agents could be a therapeutic route. With the aim to identify proteomimetics of NPM1 as enhancer of amyloid aggregation of cytoplasmatic variants, we performed bioinformatic analyses on a series of mutated peptides mimicking NPM1_264‐277_ with single and multiple point mutations. 14 single peptides were analyzed singularly in i) kinetic ThT assays, to evaluate an accelerator/delayer effect of mutations with respect to wt sequence, and in ii) CD analyses over time to speculate on different conformational transitions associated with amyloid aggregation. These studies pointed out that the sequence of NPM1_264‐277_ is very sensitive to mutations: Kinetic profiles allowed to select NPM1_264‐277_ K^267^R and NPM1_264‐277_ F^268^Y as the most modulator sequences while NPM1_264‐277_ K^267/273^ R, F^268^Y as a negative control. NPM1_264‐277_ K^267^R resulted as accelerator while NPM1_264‐277_ F^268^Y as delayer of aggregation; these effects resulted both accompanied by structural variations during time. Indeed, both sequences exhibited random → ordered structures, α + β, but with different kinetic: This transition was faster for NPM1_264‐277_ F^268^Y (t = 0.5 h) with respect to NPM1_264‐277_ K^267^R (t = 1.5 h); conversely, NPM1_264‐277_ K^267/273^ R, F^268^Y resulted unable to aggregate and structurally endowed with a greater α‐helical content with respect to the other sequences. Interestingly, NPM1_264‐277_ K^267^R and NPM1_264‐277_ F^268^Y are single mutated sequences bearing substitutions in the most APR fragment of NPM1, the stretch 267‐273.^[^ ^76^, ^77^ ^]^ In detail, the electrostatic substitution Lys ^267^/Arg in NPM1_264‐277_ K^267^R favors the self‐recognition likely for the creation of more H‐bonds in the case of the guanidine group of Arg with respect to the primary amine in Lys. Conversely, in the mutation Phe ^268^/Tyr of NPM1_264‐277_ F^268^Y, the presence of hydroxyl group of phenol of Tyr can limit preferential packing patterns of the phenyl side‐chain of Phe, since the interdigitation of the side chains between β‐sheets is crucial for ordered amyloid fold. These differences did not reflect in the morphologies of fibers which presented comparable lengths and diameter. On the other hand, in NPM1_264‐277_ K^267/273^ R, F^268^Y, the combination of abovementioned substitutions, within the amyloid core, along with the additional outside Lys ^273^/Arg, completely represses amyloid aggregation. However, to probe the efficacy of NPM1_264‐277_ K^267^R and NPM1_264‐277_ F^268^Y sequences as potential enhancers of amyloid aggregation, we employed them as seeds in ASA experiments with respect to NPM1_264‐277_ and Cterm_mutA. In this context, NPM1_264‐277_ K^267^R demonstrated to accelerate the aggregation of amyloids as confirmed by SEM analysis, likely inserting among β‐sheet aggregates oriented in antiparallel manner. This study clearly pointed out the possibility to design protein fragments to enhance amyloidogenic propensity exhibited by wt sequence through oriented and conservative mutations and confirmed the selective vulnerability and responsivity of OCI‐AML3 in stress conditions to cytotoxic effects. In conclusion, in personalized‐medicine approaches to target NPM subgroup of AML, the present study could pave the way to future and innovative therapies that selectively target leukemic cells. Even if further and in more, in‐depth cellular assays concerning the entire NPM1 interactome are required to translate the described approach into a therapeutic route.

## 4 Experimental Section

4.1

4.1.1

##### Peptide Synthesis

NPM1_264‐277_ derived peptides were synthesized as already reported,^[^ ^78^ ^]^ while Cterm_mutA (QESFKKQEKTPKTPKGPSSVEDIKAKMQASIEKGGSLPVEAKFINYVKNCFRMTDQEAIQVLCLAVEEVSLRK) was purchased from NovoPro Bioscience Inc. (Shanghai, China).

All peptides were treated with 1,1,1,3,3,3‐hexafluoro‐2‐propanol (HFIP) to guarantee a monomeric state, lyophilized and stored at−20 °C until use.

##### Far‐UV CD spectroscopy

Samples were prepared by dilution of freshly prepared stock solutions (1 mM peptide on average). CD spectra were recorded on a Jasco J‐815 spectropolarimeter (JASCO, Tokyo, Japan) at 25 °C in the far‐ultraviolet (UV) region from 190 to 260 nm. Other experimental settings were as follows: 20 nm min^−1^ scan speed, 2.0 nm band width, 0.2 nm resolution, 50 mdeg sensitivity, and 4s response. Each spectrum was obtained averaging three scans, subtracting contributions from corresponding. Peptides concentration was 200 μM in 10 mM borate buffer pH 8.5 in a 0.1 cm path‐length quartz cuvette. The measurements were recorded until 24 h. Deconvolutions of CD spectra were obtained by BESTSEL software (http://bestsel.elte.hu/).^[^ ^79^ ^]^

##### Fluorescence Assay

ThT fluorescence assays were carried out at a concentration of 200 μM for all peptides in borate buffer 50 mM with ThT 50 μM at 25 °C on the fluorescence reader Envision 2105 (Perkin Elmer) in black plates (96 well) under stirring. ASA: NPM1_264‐277_ K^267^R and NPM1_264‐277_ F^268^Y were pre‐aggregated for 4 h at 400μM in borate buffer 50 mM pH 8.5. Seeded NPM1_264‐277_ K^267^R and NPM1_264‐277_ F^268^Y were added to NPM1_264‐277_ monomer (200 μM) at a final concentration of 40 μM or to Cterm_mutA monomer (80 μM) at a final concentration of 16 μM, in both cases seed: monomer 1:5 molar ratio. Measurements were collected every 8 min up to 135 min for NPM1_264‐277_ and 6 h for Cterm_mutA.

*t*_1/2_ values were evaluated following the fitting of data of *F* emission versus time through the empirical Hill equation^[^ ^80^ ^]^ as follows.

$$F(t)=F_{\max}\frac{{\left(t/t_{1/2}\right)}^{n}}{1+{\left(t/t_{1/2}\right)}^{n}}$$

where *F*(*t*) is the fluorescence intensity at time *t*, *F*_max_ is the fluorescence intensity, and n is a cooperativity parameter.

##### DLS assay

Cterm_mutA (140 μM) in the presence of NPM1_264‐277_ K^267^R (28 μM) as seed at the ratio of 1:0.2 was kept under stirring in 50 mM phosphate, pH = 7.4, at 25 °C. The measurements were performed using a Zetasizer Nano S DLS device from Malvern Instruments (Malvern, Worcestershire, UK) with 633 nm laser, backscatter angle of 173° mode, thermostated with a Peltier system, and using a plastic micro cuvette. Size distributions by intensity were determined in automatic mode at regular time intervals over a period of 10 min for each measurement. Thirteen acquisitions were recorded, each of 10 s in duration.

##### FT‐IR

Fourier transform infrared (FT‐IR) spectra of Cterm_mutA and Cter_mutA + NPM1_264‐277_ K^267^R as seed, using the solutions analyzed in the DLS assay, were recorded on a Jasco FT/IR 4100 spectrometer (Easton, MD, USA). Measurements were performed with a Ge single crystal at a resolution of 4 cm^−1^ by drying the samples under vacuum. For each sample, 100 scans were recorded at a rate of 2 mm‐s^−1^ against a KBr background. After collection in transmission mode, the spectra were converted to emission. Amide I deconvolutions were automatically returned as emission with the integrated software (Spectra Manager 2.5).

##### SEM analysis

For the preparation of samples, NPM1_264‐277_ (200 μM), Cterm_mutA (80 μM) and NPM1_264‐277_ analogs (NPM1_264‐277_ K^267^R, NPM1_264‐277_ K^267/273^ R, NPM1_264‐277_ K^267/273^ R, F^268^Y; NPM1_264‐277_ F^268^Y; NPM1_264‐277_ F^268^Y, Y^271^F and NPM1_264‐277_ F^268/276^Y) (200 μM) were dissolved in borate buffer 5 mM and placed under stirring. After 4 h (for NPM1_264‐277_ and mutated peptides) and 48 h (for Cterm_mutA), 50 μL of aggregate solutions were placed on an aluminum stub, and the solvent was allowed to evaporate. Concerning seeding experiments, NPM1_264‐277_ (200 μM) + seeded NPM1_264‐277_ K^267^R (40 μM) and Cterm_mutA (80 μM) + seeded NPM1_264‐277_ K^267^R (16 μM) were analyzed at t = 4 h and t = 48 h of aggregation, respectively. Samples were analyzed in by using two different microscopes and were preliminary sputtered with Au/Pd conductive layer using a Denton Vacuum Desk V TSC coating system and an Agar Sputter coater respectively. SEM micrographs were recorded with the following instruments: a) field emission gun SEM (FEG‐SEM) FEI/Thermofisher Nova NanoSem 450, at 3.00 kV in high vacuum mode, using an Everhart Thornley Detector (ETD) and the through the lens detector (TLD); b) a FESEM/EDS (field emission SEM) Zeiss Merlin VP Compact coupled with a microanalysis unit (Oxford Instruments) and an INCA X‐Max solid‐state detector (Carl–Zeiss). Data sets were evaluated by means of INCA Energy software 5.05 (XPP array and pulse pile‐up corrections) with following operative conditions: 15‐kV primary beam voltage, 50–100 A filament current, variable spot size, 50 s real‐time counting.

##### Cells

OCI‐AML3 cell lines (RRID:CVCL_1844) are a kind gift of Prof E. Colombo University of Milan. They were grown at 37 °C in a humidified atmosphere of 5% CO_2_ in Modified Eagle Medium (MEM) (Microgem) supplemented with 20% heat inactivated fetal bovine serum (FBS) (Microgem) and 2 mM l‐glutamine, 50 ng/mL streptomycin, and 50 units/mL penicillin. To evaluate genotoxic stress conditions,^[^ ^81^ ^]^ cells were pretreated for 2 h with H_2_O_2_ (1 mM) and left at 37 °C in a humidified atmosphere of 5% CO_2_. After incubation, cells were washed and used for viability assays.

##### Cell Viability Assay

OCI‐AML3 cells were preincubated (2 h) with hydrogen peroxide (H_2_O_2_) 1 mM or left untreated. After the incubation, cells were washed and seeded in triplicates in 96‐well plates at a density of 5000 cells/well. NPM1_264‐277_ K^267^R, NPM1_264‐277_ K^267/273^ R, F^268^Y, NPM1_264‐277_ peptides alone (400 μM), and in combination for the sample ASA (NPM1_264‐277_ (400 μM) + NPM1_264‐277_ K^267^R (80 μM)) were incubated in 50 mM borate buffer (pH 8.5) and stirred. At two different stirring times, t = 0 and t = 24 h, they were added to the cell cultures in triplicate at a fourfold dilution of the peptide concentration and left for 24 h. During the last 4 h of incubation, 3‐(4,5‐dimethylthiazol‐2‐yl)‐2,5‐diphenyl‐2 H‐tetrazolium bromide (MTT) was added to the cells. Dimethyl sulfoxide (DMSO) was added to allow MTT to be reduced to formazan crystals by live cells. Absorbance was measured at 560 nm with the Glomax Discover microplate reader (Promega Madison, WI, USA).

##### Statistical Analysis

Statistical analysis was performed for the MTT assay. Data are expressed as the mean ± standard deviation (SD) from one experiment performed in triplicate. To evaluate statistical significance, two‐way analysis of variance (ANOVA) was used to evaluate differences among groups, considering two independent variables simultaneously. Dunnett's post hoc test was applied for multiple comparisons against the control group to adjust for type I error that was applied.

A *p* value (*p* < 0.0001) was considered indicative of statistical significance.

All analyses were carried out using GraphPad Prism 9 software.

## Conflict of Interest

The authors declare no conflict of interest.

## Supporting information

Supplementary Material

## Data Availability

Data openly available in a public repository that issues datasets with DOIs.
