# Supplementary material for: Peptidomimetics Targeting the Amyloidogenicity of Nucleophosmin 1 Mutations in Acute Myeloid Leukemia
Source: Chembiochem. 2025 Jun 17;26(14):e202500306. doi: 10.1002/cbic.202500306 (PMC12278350; doi:10.1002/cbic.202500306)
Supplement: Supplementary file 1 — Supplementary Material [file CBIC-26-e202500306-s001.pdf]

Peptidomimetics Agents Targeting the Amyloidogenicity of Nucleophosmin 1 Mutations in Acute Myeloid Leukemia

Daniele Florio[a], Sara La Manna[a], Giada Raffaella Fiore[a], Valentina Roviello[b], Giuliano Castellano[c], Sossio Fabio Graziano[a], Anna Maria Malfitano[c] and Daniela Marasco[a],*

[a] Dr. D. Florio, Dr. S. La Manna, Ms G. R. Fiore, Prof. S. F. Graziano, Prof. D. Marasco
Department of Pharmacy
University of Naples “Federico II”
80131, Naples, Italy
*E-mail: daniela.marasco@unina.it

[b] Dr. V. Roviello
Department of Agriculture
University of Naples “Federico II”
80055, Naples, Italy

[c] Dr. G. Castellano, Prof. A. M. Malfitano
Department of Translational Medical Science
University of Naples "Federico II"
80131 Naples, Italy

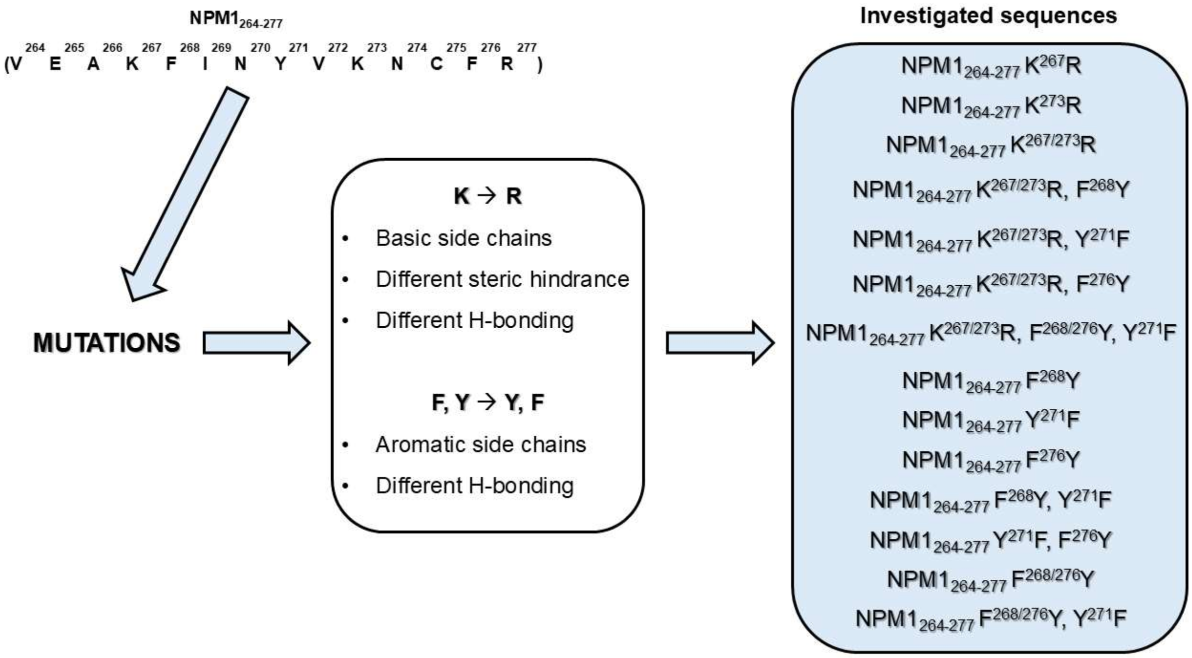

Fig. S1. Schematic overview of the peptide design of NPM1$_{264–277}$ mutated sequences examined in this study.

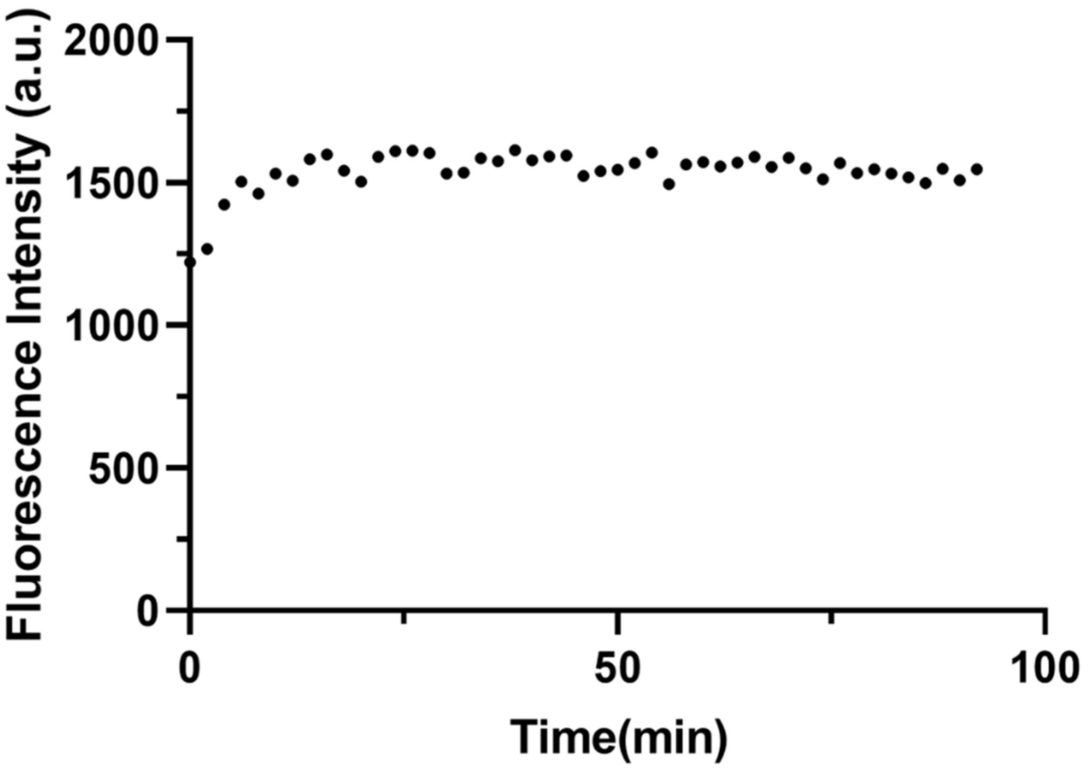

Fig. S2. Time course of ThT fluorescence emission intensity of $NPM1_{264\text{-}277}$ sequence.

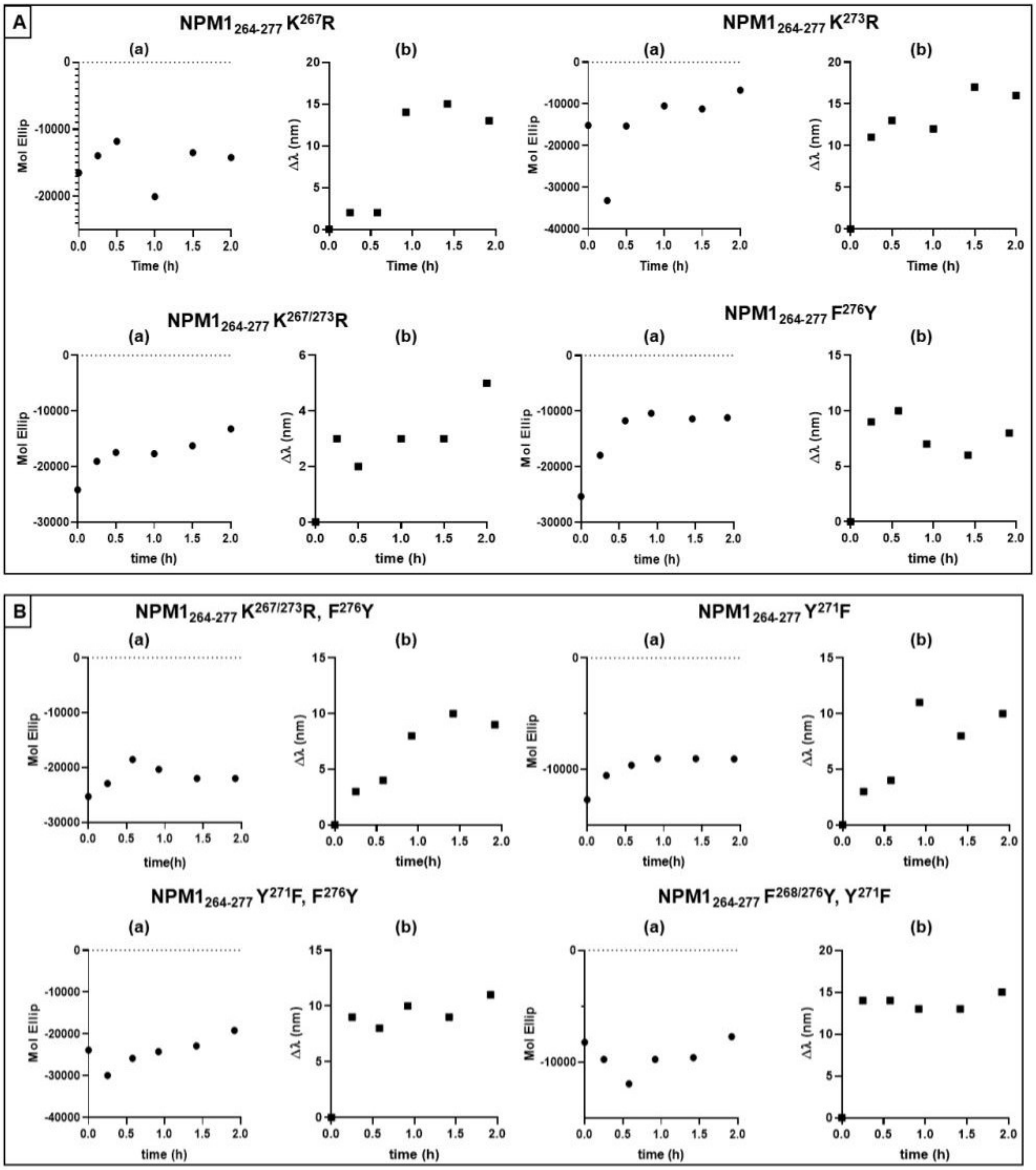

Fig. S3. NPM1$_{264\text{-}277}$ analogues: (A) NPM1$_{264\text{-}277}$ K^{267}R - NPM1$_{264\text{-}277}$ K^{273}R - NPM1$_{264\text{-}277}$ K$^{267/273}$R - NPM1$_{264\text{-}277}$ F^{276}Y, (B) NPM1$_{264\text{-}277}$ K$^{267/273}$R, F^{276}Y - NPM1$_{264\text{-}277}$ Y^{271}F - NPM1$_{264\text{-}277}$ Y^{271}F, F^{276}Y - NPM1$_{264\text{-}277}$ F$^{268/276}$Y, Y^{271}F. a) Minima of Mol Elli *vs* time and b) $\Delta\lambda_{min}$ (=λ_{ti}-λ_{t0}) vs time.

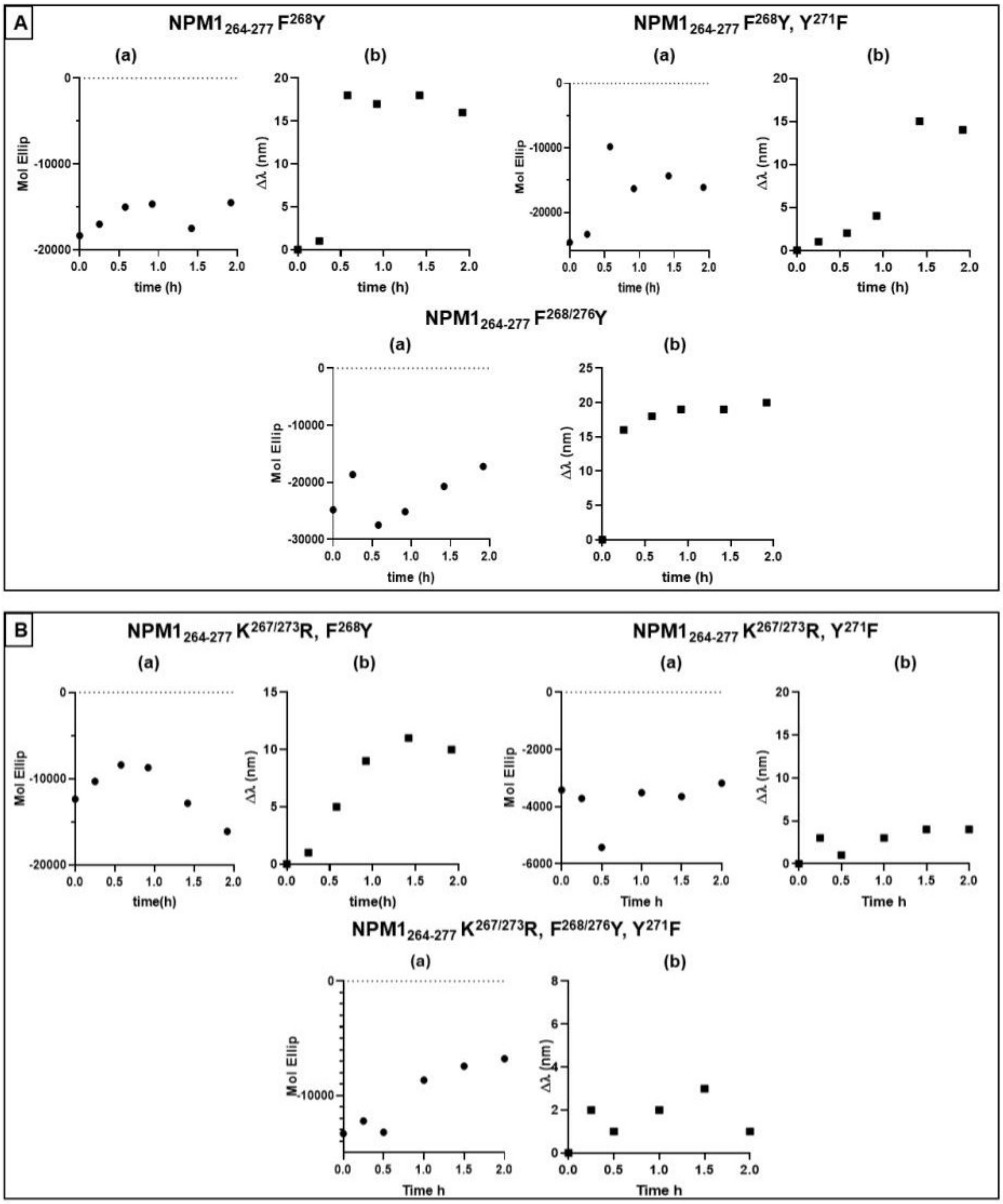

Fig. S4. NPM1$_{264\text{-}277}$ analogues: (A) NPM1$_{264\text{-}277}$ F^{268}Y - NPM1$_{264\text{-}277}$ F^{268}Y, Y^{271}F - NPM1$_{264\text{-}277}$ F$^{268/276}$Y, (B) NPM1$_{264\text{-}277}$ K$^{267/273}$R, F^{268}Y - NPM1$_{264\text{-}277}$ K$^{267/273}$R, Y^{271}F - NPM1$_{264\text{-}277}$ K$^{267/273}$R, F$^{268/276}$Y, Y^{271}F. a) Minima of Mol Elli *vs* time and b) $\Delta\lambda_{min}$ (=λ_{ti}-λ_{t0}) vs time.

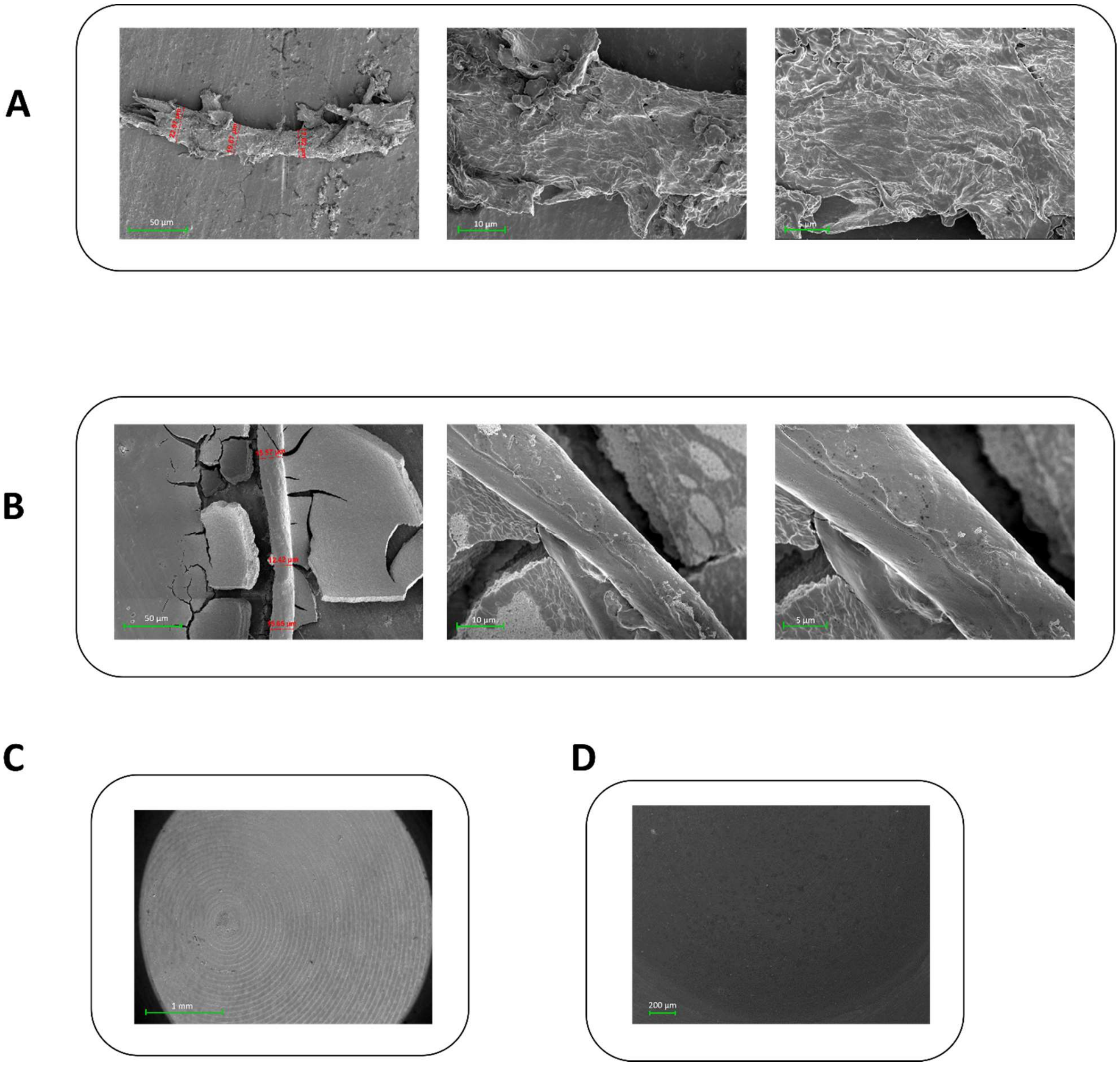

Fig. S5. SEM micrographs of of (A) NPM1$_{264\text{-}277}$ alone (200 µM), (B) Cterm_mutA alone (80 µM), (C) NPM1$_{264\text{-}277}$ K$^{267/273}$R, F^{268}Y alone and (D) NPM1$_{264\text{-}277}$ F$^{268/276}$Y alone (200 µM). Overviews of the surface of samples at: 50 µm (left column), 10 µm (central column) and 5 µm (right column) for A-B panels; 1mm and 200 µm for C-D panels respectively.

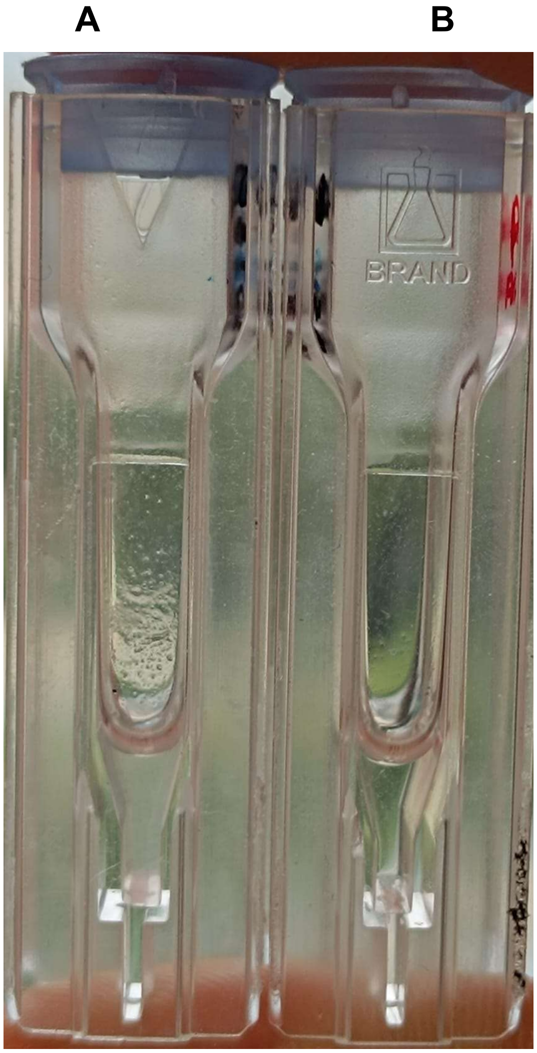

Fig. S6. Image of samples (A) Cterm_mutA + NPM1$_{264-277}$ K^{267}R as seed at the ratio of 1:0.2 and (B) Cterm_mutA alone in 50mM phosphate buffer after 2h of stirring. In the presence of NPM1$_{264-277}$ K^{267}R the formation of insoluble aggregates is observed.

Table S1: Deconvolution of CD spectra of selected $NPM1_{264\text{-}277}$ analogues at indicated times.

		Time (h)	α-helix	β-sheet	Turn	Others
Group A	$NPM1_{264\text{-}277}$ $K^{267}R$	0	6.4	25.2	19.3	49.0
		0.5	9.2	22.7	16.9	51.2
		1	32.7	25.3	0.4	41.6
		1.5	15.6	36.5	7.1	40.8
		2	18.5	35.0	6.7	39.8
	$NPM1_{264\text{-}277}$ $K^{273}R$	0	4.8	24.7	18.0	52.5
		0.5	22.9	22.1	7.9	47.1
		1	12.4	33.9	9.8	43.9
		1.5	12.4	33.9	9.8	43.9
		2	6.2	30.9	11.8	50.9
	$NPM1_{264\text{-}277}$ $K^{267/273}R$	0	18.7	27.1	11.4	42.8
		0.5	25.6	22.5	9.2	42.7
		1	6.0	33.3	13.0	47.9
		1.5	22.3	23.0	8.8	45.9
		2	4.0	39.3	13.2	43.5
	$NPM1_{264\text{-}277}$ $F^{276}Y$	0	15.4	23.3	14.3	47.0
		0.5	12.4	33.5	8.7	45.4
		1	11.0	33.1	11.6	44.3
		1.5	10.0	32.4	11.1	46.5
		2	8.9	36.7	11.1	43.3
Group B	$NPM1_{264\text{-}277}$ $K^{267/273}R$, $F^{276}Y$	0	15.0	30.3	16.7	38.0
		0.5	12.7	30.9	17.8	38.6
		1	32.3	16.4	7.0	44.4
		1.5	37.9	13.4	2.9	45.8
		2	41.4	11.4	3.7	43.5
	$NPM1_{264\text{-}277}$ $Y^{271}F$	0	4.7	27.5	18.0	49.8
		0.5	5.7	29.5	16.7	48.1
		1	12.4	28.1	13.1	46.4
		1.5	10.6	29.3	14.1	46.0
		2	10.5	27.2	14.8	47.6
	$NPM1_{264\text{-}277}$ $Y^{271}F$, $F^{276}Y$	0	14.0	25.3	14.5	46.2
		0.5	15.5	39.9	5.5	39.1
		1	12.9	42.0	6.6	38.6
		1.5	12.6	42.9	7.0	37.5
		2	21.8	33.2	2.6	42.4
	$NPM1_{264\text{-}277}$ $F^{268/276}Y$, $Y^{271}F$	0	4.3	32.2	15.9	47.6
		0.5	13.3	51.2	3.0	32.5
		1	8.7	51.6	5.6	34.1
		1.5	11.4	43.8	7.4	37.3
		2	7.2	43.5	10.2	39.0
Group C	$NPM1_{264\text{-}277}$ $F^{268}Y$	0	11.1	31.5	16.0	41.4
		0.5	6.4	31.5	14.4	47.7
		1	21.7	37.1	3.1	38.2
		1.5	24.0	37.9	4.8	33.4
		2	23.9	34.8	4.4	36.9
	$NPM1_{264\text{-}277}$ $F^{268}Y$, $Y^{271}F$	0	8.7	25.7	17.9	47.8
		0.5	13.6	20.5	16.0	50.0
		1	11.9	25.9	13.5	48.6
		1.5	27.2	38.6	0.0	34.2
		2	21.5	46.2	1.6	30.7
	$NPM1_{264\text{-}277}$ $F^{268/276}Y$	0	0.7	37.6	17.1	44.6
		0.5	15.3	53.6	1.9	29.2
		1	12.3	54.8	2.7	30.1
		1.5	20.8	51.4	0.0	27.8
		2	18.6	49.3	2.2	30.0

**Group D**	NPM1$_{264\text{-}277}$ K$^{267/273}$R, F^{268}Y	0	1.9	31.4	19.4	47.3
		0.5	0.6	28.2	21.0	50.3
		1	12.8	33.5	11.7	42.0
		1.5	20.3	25.0	11.2	43.4
		2	27.6	21.3	7.5	43.5
	NPM1$_{264\text{-}277}$ K$^{267/273}$R, Y^{271}F	0	9.7	81.2	16.4	52.7
		0.5	13.9	22.4	16.6	47.1
		1	7.4	28.1	15.2	49.2
		1.5	7.9	28.8	14.7	48.5
		2	8.4	34.2	14.4	43.0
	NPM1$_{264\text{-}277}$ K$^{267/273}$R, F$^{268/276}$Y, Y^{271}F	0	7.8	28.8	16.4	47.0
		0.5	8.9	29.3	16.2	45.5
		1	8.4	29.3	14.9	47.4
		1.5	4.3	34.2	14.6	46.8
		2	4.5	35.5	14.4	45.6
